# NOX2-Deficient Neutrophils Facilitate Joint Inflammation Through Higher Pro-Inflammatory and Weakened Immune Checkpoint Activities

**DOI:** 10.3389/fimmu.2021.743030

**Published:** 2021-09-07

**Authors:** Yi-Chu Liao, Szu-Yu Wu, Ya-Fang Huang, Pei-Chi Lo, Tzu-Yi Chan, Chih-An Chen, Chun-Hsin Wu, Che-Chia Hsu, Chia-Liang Yen, Peng-Chieh Chen, Chi-Chang Shieh

**Affiliations:** ^1^Institute of Clinical Medicine, College of Medicine, National Cheng Kung University, Tainan, Taiwan; ^2^National Laboratory Animal Center, National Applied Research Laboratories, Tainan, Taiwan; ^3^Laboratory of Innate Immune Systems, Department of Microbiology and Immunology, Graduate School of Medicine, Osaka University, Osaka, Japan; ^4^Department of Pediatrics, National Cheng Kung University Hospital, Tainan, Taiwan; ^5^Division of Allergy, Immunology and Rheumatology, Department of Internal Medicine, National Cheng Kung University Hospital, College of Medicine, National Cheng Kung University, Tainan, Taiwan; ^6^Department of Orthopedic Surgery, National Cheng Kung University Hospital, College of Medicine, National Cheng Kung University, Tainan, Taiwan

**Keywords:** NOX2, chronic granulomatous disease, serum-induced arthritis, neutrophils, immune checkpoint, reactive oxygen species

## Abstract

Immune-mediated arthritis is an important chronic inflammatory disease of joints causing debilitating morbidity in affected patients. The mechanisms underlying immune-mediated arthritis have been intensively investigated, however the cellular and molecular factors contributing to the joint inflammation in different redox conditions have not been clearly elucidated. Previous research showed that phagocyte-produced reactive oxygen species (ROS) plays an anti-inflammatory role in K/BxN serum-transfer arthritis and NOX2-deficient mice tend to have more severe arthritis. Although many leukocytes play critical roles in the development of immune-mediated arthritis, the role of neutrophils, which are the main producers of ROS in inflammation, is still controversial. We hence assessed the immunomodulatory function of neutrophils from arthritic joints of NOX2-deficient and wild type mice in this study. We found more neutrophils accumulation in NOX2-deficient inflamed joints. RNA-sequencing and quantitative PCR revealed significantly increased expression of acute inflammation genes including *IL1b, Cxcl2, Cxcl3, Cxcl10* and *Mmp3* in activated neutrophils from the inflamed joints of NOX2-deficient mice. Moreover, gene set enrichment analysis (GSEA) showed enriched gene signatures in type I and II IFN responses, IL-6-JAK-STAT3 signaling pathway and TNF-α signaling pathway *via* NF-κB in NOX2-deficient neutrophils. In addition, we found that NOX2-deficient neutrophils expressed lower levels of PD-L1 and were less suppressive than WT neutrophils. Moreover, treatment of PD-L1-Fc decreased cytokine expression and ameliorated the severity of inflammatory arthritis. Our results suggest that NOX2-derived ROS is critical for regulating the function and gene expression in arthritic neutrophils. Both the strong pro-inflammatory and weakened anti-inflammatory functions of neutrophils due to abnormal redox regulation may be targets of treatment for immune-mediated arthritis.

## Introduction

Immune-mediated inflammatory arthritis, including rheumatoid arthritis (RA) and juvenile idiopathic arthritis, is a family of chronic inflammatory diseases of joints characterized by tissue inflammation, synovial proliferation, autoantibody production and destruction of cartilage and bones (1). Clinical and laboratory investigations have demonstrated that inflammatory cytokines including tumor necrosis factor-α (TNF-α), interleukin 6 (IL-6), interleukin 1β (IL-1β) and granulocyte-monocyte colony stimulating factor (GM-CSF) contribute to this inflammatory process (2). Synovial inflammation occurs when leukocytes, including innate and adaptive immune cells, infiltrate into the joint compartment (3, 4). The types and functions of immune cells participating in the initiation and progression of different types of joint inflammation, however, are more difficult to be determined. Neutrophils in arthritis are active in the coordinating progress of inflammation by regulating the functions of other immune cells. Many activated neutrophils are found in synovial fluid of arthritis and have a potential to cause serious damage within inflamed joints (5, 6). Neutrophils are the first line of host defense against a wide range of infectious pathogens (7). They are also terminally differentiated, relatively short-lived leukocytes that can communicate with adaptive immune cells through cytokine secretion and cell-cell interaction (8–10). The role and function of neutrophils in the initiation stage of joint inflammation attracted our attention. Understanding the function and gene expression profile of neutrophils in immune-mediated arthritis is important for developing therapeutic interventions.

Both NADPH oxidase and mitochondria contribute significantly to intracellular ROS generation, however, phagocytes-expressed NOX2-derived ROS plays important role in immune-mediated inflammation. When activated, NOX2 generates high concentrations of ROS in the phagosome to kill the engulfed microbes (11). Excessive ROS production are associated with several types of inflammation. However, more and more evidences suggest that ROS serve as signaling intermediates that play important roles in several pathways, and also as significant regulators of immune cells (12). Inherited mutations in the subunits of NOX2 result in chronic granulomatous disease (CGD), a primary immunodeficiency disease, and patients with CGD experience fail host defense, abnormal granuloma formation and recurrent infection (13). Also, several previous studies have pointed to an association of CGD with autoimmune arthritis (14). It has been shown that the mutated *Ncf1*, a gene encoded the p47 phox subunit of NOX2 complex, led to a more severe collagen-induced arthritis (15, 16). Numbers of diverse autoimmune conditions in the CGD population have been observed, suggesting that patients with CGD are at an increased risk for developing autoimmune disorders (13, 17, 18).

K/BxN serum-transfer arthritis model is induced by transferring serum from K/BxN mice to naive mice. The manifestations of arthritis occur several days later. The inflammatory response is driven by autoantibodies against glucose-6-phosphate isomerase (G6PI), leading to the formation of immune complexes that drive the activation of different innate immune cells such as neutrophils and macrophages (19). It can be induced in a wide range of strains, including specific gene-deficient mice, to study the specific significance of genes. This murine arthritis model displays many characteristics that similar to human RA, such as leukocyte infiltration, synovitis, pannus formation, and cartilage and bone erosion (20). As we are interested in the role of innate immune cells in the initial stage of arthritis, K/BxN serum-transfer arthritis model is an ideal model to study the effector mechanisms involved in progression of disease.

In our previous studies, NOX2-deficient mice acquired more severe K/BxN serum-transfer arthritis (21). Here, we further characterized the role of NOX2-deficient, serum-activated neutrophils from the inflamed joints to investigate the mechanistic roles of neutrophils in the redox regulation of immune-mediated arthritis.

## Materials and Methods

### Mice

K/BxN mice were generated from breeding KRN TCR transgenic mice on a C57BL/6 background (a gift from Dr. D. Mathis and Dr. C. Benoist, Harvard Medical School, Boston, MA, and the Institut de génétique et de biologie moléculaire et cellulaire, Strasbourg, France) with NOD mice (purchased from the National Laboratory Animal Center, Taiwan) (19). Mice deficient in *Ncf1* [B6(Cg)-Ncf1m1J/J, No. 004742] were purchased from Jackson Laboratory. C57BL/6 mice purchased from National Laboratory Animal Center were used as WT mice. Male mice, aged 10–12 weeks, were used for experiments. Three to five mice were used for each group, and each experiment was repeated 2-3 times. All mice have been routinely backcrossed to C57BL/6 background, undergone genome-wide genotyping to confirm the genetic background, and housed in the animal facility of the Laboratory Animal Center at National Cheng Kung University (21). All experimentation protocols were approved by the Institutional Animal Care and Use Committee (IACUC), National Cheng Kung University.

### K/BxN Serum-Transfer Arthritis

Serum was collected from K/BxN mice at 8-9 weeks of age and stored at -80°C. About 250 µl serum was acquired from each K/BxN mouse, and approximately 2 mL serum was collected from 5-8 K/BxN mice in one batch. Serum was mixed from different batches and 100 µl serum plus 50 µl PBS were injected into one mouse for induction on day 0 and again on day 2 (21, 22). Clinical scores were analyzed as the sum of the four limbs, and which would be given a score of 0-3 per limb. 0, no observable swelling; 1, one or two involved digits or mild swelling of the larger structures of the wrist, foot, and ankle, but where the foot shows its normal V shape; 2, between one and three, the long edges of the foot are parallel to each other with disappearance of the original V shape; and 3, severe arthritis, the wrist shows swelling extending along the dorsum of the paw to the base of the digits, and the ankle shows the inversion of the V shape by expansion of the ankle and hind foot to greater than the width of the fore foot often accompanied by digital swelling. Joint swelling were quantitated as the change in thickness of all four paws as measured with a caliper (Peacock dial thickness gauge with flat anvils; Ozaki Mfg. Co., Ltd., Tokyo, Japan). The measurements were performed before injection on day 0 and once a day after injection from day 1 to day 7 when the mice were sacrificed (21, 22). Ankle and wrist tissues were collected for neutrophil isolation. For PD-L1-Fc treatment, recombinant PD-L1-Fc (BioLegend, catalog No. 758208) was i.p. (20 mg/mouse/day) injected into induced *Ncf1^-/-^* mice at day 0, 2, 4, 6.

### Flow Cytometry

Cells were blocked with purified rat anti–mouse CD16/CD32 Fc block (BD Biosciences, catalog No.553142) at 4°C for 15 minutes, prior to incubation with various fluorescence-conjugated antibodies at 4°C for 30 minutes. Cells were washed twice after antibody incubation. A single-stained control for each fluorochrome was made for compensation for each experiment. Labeled cells were detected by flow cytometry with FACS Calibur (BD Bioscience) or FACS Canto II instruments (BD Biosciences) and analyzed with FlowJo software (TreeStar). Mouse Gr-1 FITC conjugated antibody (Invitrogen, REF. No. RM3001), APC conjugated anti-mouse Ly6G antibody (BioLegend, catalog No.127613), PerCP/Cyanine5.5 conjugated anti-mouse CD274 antibody (PD-L1; BioLegend, catalog No.124333) were used.

### Isolation of Neutrophils From Joints

Mice joints were dissected and shattered by scissor, and minced tissue were immersed in medium containing 50ug/ml Liberase (Roche, catalog No. 05401127001) and 1ug/ml Dnase I (Sigma, catalog No. D4527), and then 37°C incubation for 45 mins in shaker. Minced tissue were then grinded, and cell suspension were filtered, and then were separated by using percoll (GE, catalog No.17089101) and centrifugation. Neutrophils were sorted out by using biotin conjugated anti-Gr-1 antibody (BD Bioscience, catalog No.553125) and biotin conjugated anti-Ly6G antibody (BioLegend, catalog No. 127603), Streptavidin MicroBeads (Miltenyl Biotec, catalog No. 130-048-102), LS column and MACS (Miltenyl Biotec, catalog No. 130-042-401) (23).

### Splenic T Cell Isolation and Proliferation Assay

T cells from the mouse spleen were isolated by using depleting anti-bodies, including anti-mouse CD16/32 (BD Pharmingen, catalog No.553142), biotin anti-mouse CD11c (BD Pharmingen, catalog No.553800), biotin anti-mouse FcεRIα(BioLegend, catalog No.134304), biotin anti-mouse NK1.1(BD Pharmingen, catalog No.553183), biotin anti-mouse CD19 (BD Pharmingen, catalog No.553784), biotin anti-mouse TER119 (BioLegend, catalog No. 116204), biotin anti-mouse F4/80 (BioLegend, catalog No. 123105) and biotin anti-mouse Ly-6G and Ly-6C (BD Pharmingen, catalog No.553125), Anti-Biotin MicroBeads UltraPure, LS column and MACS (Miltenyl Biotec, catalog No.130-042-401). Magnetically labeled cells to be excluded were retained by the magnetic field, while unlabeled T cells pass through and were collected for CFSE (BD, material No. 565082) labeling. Purified T cells were in RPMI supplemented with 10 ml of 0.05 μM β-mercaptoethanol, and 100 U/ml penicillin/streptomycin with 10% heat-inactivated FBS. To stimulate proliferation, T cells were cultured in presence of 0.1 µg/mL anti-CD3/CD28 (BD Pharmingen, catalog No.553057/553294). T cell proliferation were assayed after co-culturing with isolated neutrophils for 3 days and the ratio of neutrophils to T cells were 1:10. The CFSE content in T cells was analyzed by flow cytometry in which low CFSE labeling indicated more proliferation and high-intensity CFSE indicated less proliferation (24).

### RNA-Sequencing

RNA was extracted from approximately 1x10^5^ sorted Ly6G^+^ neutrophils with RNeasy Micro Kit (QIAGEN, REF. No. 74004), and cDNA libraries were constructed with SMART-Seq^®^ v4 Ultra^®^ Low Input RNA Kit for Sequencing (TAKARA, catalog No. 634895) followed by Nextera XT DNA Library Prep Kit (Illumina, REF. No. 15032350). Concentration of libraries were quantified with Qubit (Thermo Fisher, REF. No. Q32854) and sequenced with 2x75 pair-end sequencing on Illumina NextSeq 500 platform. Sequences were aligned to Mus musculus reference genome GRCm38 with *HISAT2* and reads of genes were counted by *HTSeq*. Differential gene expression was analyzed with *DESeq2*. Gene expression level with log2 fold change ≧1 or ≦-1 and an adjusted p-value <0.05 when comparing *Ncf1^-/-^* arthritic neutrophils with WT neutrophils were included in further analyses. The data has been deposited to https://www.ncbi.nlm.nih.gov/bioproject/PRJNA753258.

### Quantitative Real-Time PCR

Ly6G^+^ neutrophils were resuspended in Trizol reagent (Invitrogen, catalog No.15596018) and total RNA was isolated using Zymo Research Direct-zol RNA MiniPrep kit (catalog No.R2050, Irvine, CA) according to the manufacturer’s protocol. cDNA was constructed with High-Capacity cDNA Reverse Transcription Kit (Appliedbiosystems, ThermoFisher, catalog No. 4368814). Gene expression of *IL1b*, *Nos2*, *Cxcl2, Cxcl3, Cxcl10, Mmp3, Ifi27l2a, Oas2* and *Cd14* were measured by quantitative real-time PCR with transcript-specific primers using PowerUP SYBR Green Master Mix (Appliedbiosystems, ThermoFisher, Catalog No. A25741) in StepOnePlus Real Time PCR system (Appliedbiosystems, ThermoFisher). Oligonucleotide primers used to measure gene expression included:

*B-actin*,
5’-TGGAATCCTGTGGCATCCAT-3’ (forward)
5’-AAACGCAGCTCAGTAACAGT-3’ (reverse)
*IL1b*,
5’-AAGCTCTCCACCTCAATGGAC-3’ (forward)
5’-TTGGGATCCACACTCTCCAGC-3’ (reverse)
*Nos2*,
5’-CCAGGAGGAGAGAGATCCGATT-3’ (forward)
5’-GTCCATGCAGACAACCTTGG-3’ (reverse)
*Cxcl3*,
5’-CCAGACAGAAGTCATAGCCAC-3’ (forward)
5’-CGTTGGGATGGATCGCTTTTC-3’ (reverse)
*Cxcl2*
5’-CCCAGACAGAAGTCATAGCCAC-3’ (forward)
5’-TGGTTCTTCCGTTGAGGGAC-3’ (reverse)
*Cxcl10*
5’-CCACGTGTTGAGATCATTGCC-3’ (forward)
5’-GAGGCTCTCTGCTGTCCATC-3’ (reverse)
*Mmp3*
5’-GCATCCCCTGATGTCCTCGT-3’ (forward)
5’-ATTTGCGCCAAAAGTGCCTGTC-3’ (reverse)
*Ifi27l2a*
5’-CTCTGCCATAGGAGGAGCTCTG-3’ (forward)
5’-TGCACAGTGGACTTGACGGG-3’ (reverse)
*Oas2*
5’-CCTTGGAAAGTGCCAGTACCT-3’ (forward)
5’-AGCGTCTTCCAGAGCTGAAT-3’ (reverse)
*Cd14*
5’-TTGGGCGAGAGAGGACTGAT-3’ (forward)
5’-GCATCCCGCAGTGAATTGTG-3’ (reverse)

### Gene Set Enrichment Analysis (GSEA)

GSEA (www.broadinstitute.org/gsea) was used to identify molecular pathways significantly overrepresented among the upregulated and downregulated genes, and to compare the expression profiles with other published studies (25). False discovery rate (FDR) estimation is used in GSEA. Gene interacting network was display with Cytoscape (Cytoscape Consortium, http://www.cytoscape.org) (26).

### Measurement of Cytokines

Mouse wrist joints were homogenized in liquid nitrogen, ground into fine powder, then lysed with protein lysis buffer (1% Triton X-100, 150mM NaCl, 10mM Tris-base, 1mM EDTA, 1mM EGTA, pH 7.4, protease inhibitor cocktail) and homogenized on ice. After homogenization, samples were subsequently centrifuged (10 000 rpm, 20 min, 4°C) to remove debris. Samples were then frozen at −80°C for cytokine analysis. Pro-inflammatory cytokines in whole wrist homogenate samples were measured by a Milliplex mouse Th17 magnetic beads array Kit (MTH17MAG-47K, Millipore, Saint Louis, MO) in duplicate and performed with the protocols as described by the manufacturer.

### Histology

For histopathologic analysis, ankle tissues were fixed in 4% neutral buffered formalin at room temperature for 24 h and decalcified in formic acid with sodium formate for 72 h before embedded in paraffin. The paraffin tissue blocks were sectioned at 5μm on adhesive slides. The slides were deparaffinized and rehydrated before staining with anti-myeloperoxidase (MPO) antibody (Abcam, catalog No. ab9535) and H&E staining.

### Detection of Protein Carbonylation

The level of protein carbonyl groups in control and inflamed joint homogenates was determined by using Protein Carbonyl Content Assay Kit (BioVision) following the protocol provided by the manufacturer. O.D.s was measured at ~375 nm in a microplate reader.

### Detection of ROS Production

Mouse whole-blood leukocytes were washed with suspension buffer (1X HBSS and 5% FBS) three times and incubated with 10 mM H2DCF-DA at 37°C for 20 min. Cells were then incubated with 100 ng/ml PMA (Sigma-Aldrich) at 37°C for 15 min, followed by fluorescence analysis with flow cytometry (FACS Calibur, BD Biosciences).

### Statistical Analysis

The statistical differences between two groups were analyzed with Student’s t-test, and when comparing three or more groups, one-way or two-way ANOVA was used by using GraphPad Prism software version 8.0 (GraphPad Software, Inc., La Jolla, CA). p-value < 0.05 were considered significant. (*p < 0.05; **p < 0.01; and ***p < 0.001).

## Results

### Neutrophil Accumulation Was More Prominent in Serum-Induced Arthritic Joints of NOX2-Deficient Mice

**Figure 1:**
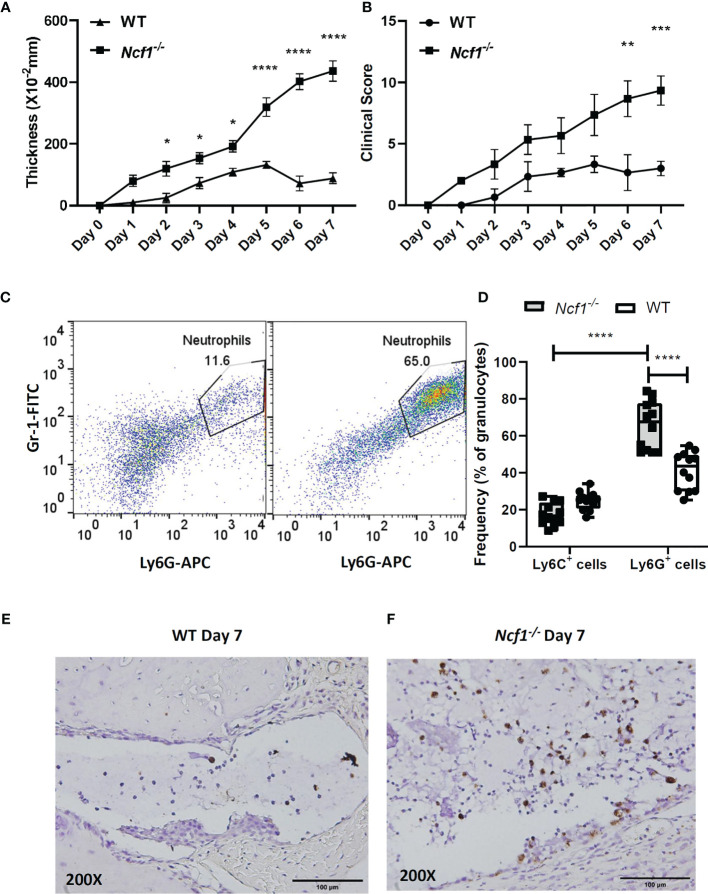
Increased neutrophil population is detected in joints of arthritic *Ncf1^-/-^* mice. The severity of arthritis was evaluated on days 0–7 with joint thickness. **(A)** and clinical scores **(B)** after the injection of K/BxN serum. The figures indicate the sum of the thickening and clinical scores of all four paws of each mouse. Asterisks indicate significant differences between *Ncf1^-/-^* mice (n=3) and WT mice (n=3). (*p < 0.05, **p < 0.01, ***p < 0.001, ****p < 0.0001). Flow cytometric analysis of the WT and *Ncf1^-/-^* **(C)** inflamed joint neutrophil population are shown. Flow cytometric analysis of Ly6G^+^ and Ly6C^+^ cell population were quantitated and are shown in **(D)**. Images of immunohistochemically staining with anti-MPO antibody are shown in **(E)** WT and **(F)** *Ncf1^-/-^* inflamed joints. The experiment was repeated twice with similar results.

In order to evaluate the role of NOX2-produced ROS in inflammatory arthritis, K/BxN serum were injected into WT and *Ncf1^-/-^* mice on day2 and day7 respectively. Swelling (thickness) and clinical scores of each mouse were measured for 7 days. We found that the joint swelling and clinical scores in *Ncf1^-/-^* arthritic mice was significantly more severe than those in WT controls (**Figures 1A, B**). In addition, neutrophil population in inflamed joints of *Ncf1^-/-^* mice was increased when compared with the inflamed joint of WT mice on day 7 (**Figure 1C**). We further analyzed the population of Ly6G^+^ and Ly6C^+^ cells. We found that the proportion of Ly6G^+^ cell was increased in the granulocyte population of *Ncf1^-/-^* inflamed joints when compared to WT control. (**Figure 1D**). In joint tissue sections stained with an MPO specific antibody, neutrophils accumulation was more prominent in *Ncf1^-/-^* joint inflammation when compared with WT controls (**Figures 1E, F**). The data were consistent with our previous findings (21). In addition, we demonstrated how NOX2 deficiency affects oxidative stress in peripheral blood leukocyte and joints. Our results showed that the ROS production of NOX2-deficient peripheral blood leukocytes is lower after PMA stimulation *in vitro* (**Supplementary Figure 1**). Moreover, protein carbonylation, an oxidative stress marker, is also decreased in NOX2-deficient joints (**Supplementary Figure 1**). These results suggest that abundant neutrophil population in arthritic joints may play a role in serum-induced arthritis of *Ncf1^-/-^* mice, we hence went on to analyze the inflammatory characteristics of the neutrophils in the arthritic tissues.

### Activated NOX2-Deficient Neutrophils Showed Strong Pro-Inflammatory Transcriptional Profiles

**Figure 2:**
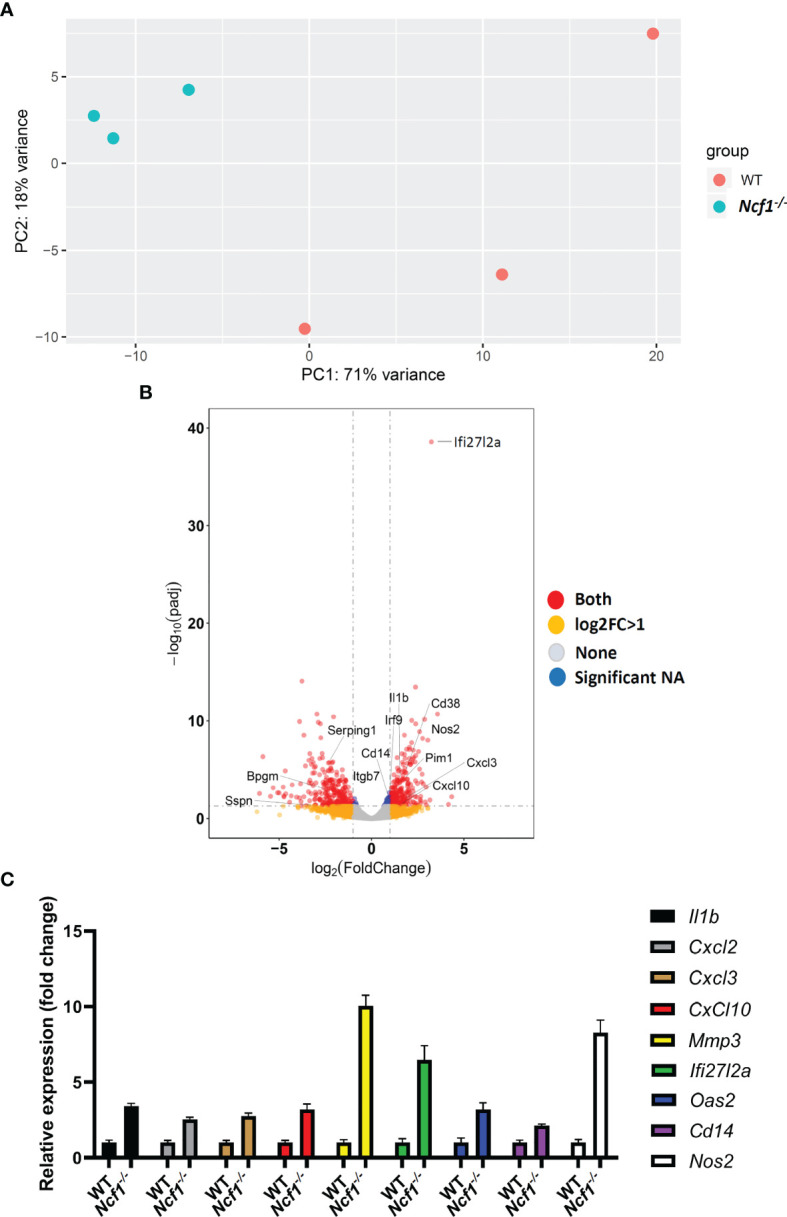
Transcriptional profiles of immune complex activated WT and NOX2-deficient neutrophils. **(A)** RNA-seq was performed on sorted Ly6G^+^ neutrophils from arthritic joints of *Ncf1^−/−^* mice (blue) (n = 3) and WT mice (red) (n = 3). **(B)** Volcano plot of differential gene expression between WT and *Ncf1^−/−^* arthritic neutrophils. Up and down regulated genes with fold change ≥1 and P < 0.05 are in red. **(C)** *IL1b*, *Cxcl2*, *Cxcl3, Cxcl10, Mmp3, Ifi27l2a, Oas2, Cd14 and Nos2* expressions were measured with qPCR, normalized to *b-actin* gene expression.

**Figure 3:**
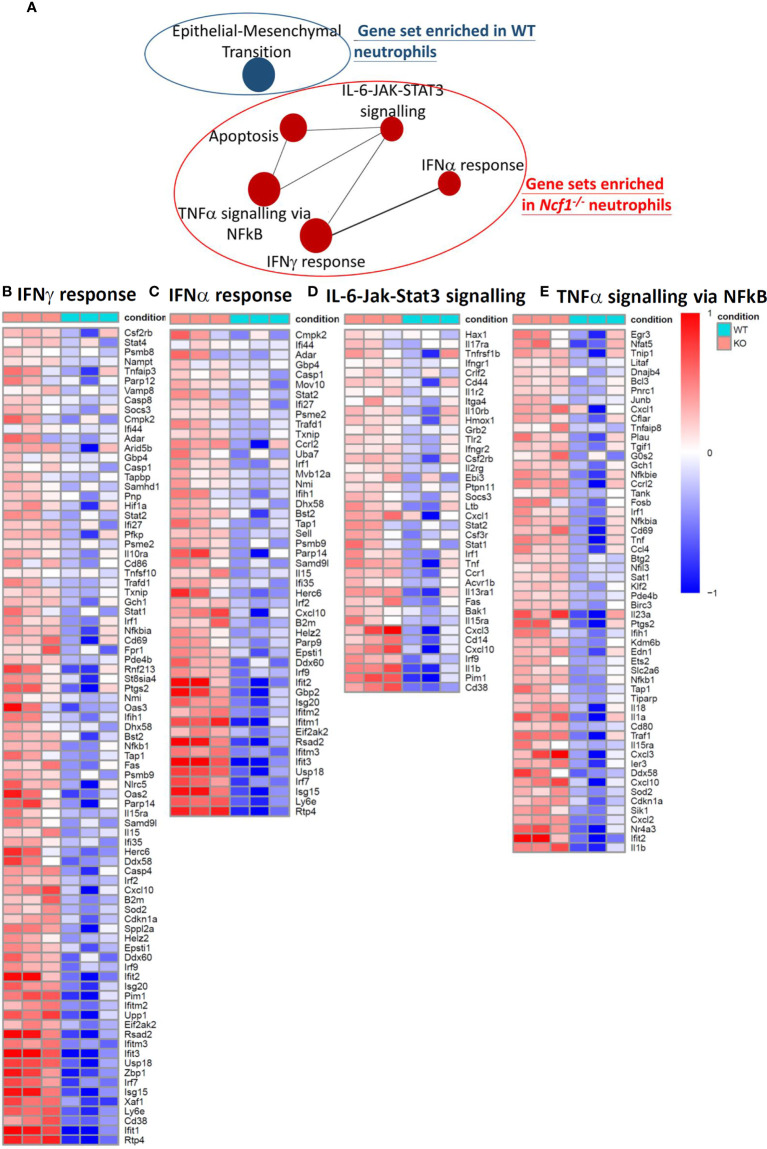
GSEA and network analysis revealed five gene sets enriched in *Ncf1^-/-^* neutrophils and one gene set enriched in WT neutrophils. **(A)** Enrichment map for differentially expressed genes in the arthritic neutrophils between *Ncf1^-/-^* and WT was generated with the Enrichment Map Plugin34 for Cytoscape program (FDR q-value < 0.25, Edge Cutoff = 0.15). Node size is proportional to the total number of genes in each gene set. The proportion of shared genes between gene sets is represented by the thickness of the line between nodes. Heat maps of RNA-seq data show up-regulated genes of **(B)** IFNγ response **(C)** IFNα response **(D)** IL-6/JAK/STAT3 signaling and **(E)** TNFα signaling *via* NF-κB from immune complex activated *Ncf1^-/-^* neutrophils.

It has been reported arthritis was abrogated when accumulated neutrophils were depleted (21) or their migration were inhibited (27), the arthritic neutrophils, especially those with NOX2 deficiency, appear to have strong pro-inflammatory activities. To investigate the global gene expression of neutrophils, we isolated Ly6G^+^ cells from the joints of WT and *Ncf1* ^-/-^ arthritic mice by using MACS, and then performed RNA-sequencing. Given the fact that limited amount of RNA were acquired, sequencing libraries of mRNA were constructed by using SMART-seq protocol. Gene expression profile of activated *Ncf1^-/-^* neutrophils distinct from that of activated WT neutrophils, including 318 up-regulated and 287 down-regulated genes (**Figure 2A**). Moreover, most of up-regulated genes in *Ncf1^-/-^* arthritic neutrophils were related to IL-6-Jak-Stat3 signaling pathway and type I, II IFN responses (**Figure 2B**). We further validated RNA-seq data by detecting cytokine gene *IL1b* (*Ncf1^-/-^* 3.41-fold increase), chemokine genes *Cxcl2 (Ncf1^-/-^* 2.53-fold increase)*, Cxcl3* (*Ncf1^-/-^* 2.75-fold increase)*, Cxcl10* (*Ncf1^-/-^* 3.18-fold increase), matrix metalloproteinase gene *Mmp3* (*Ncf1^-/-^* 10.03-fold increase), gene of interferon, alpha-inducible protein 27 like 2A *(Ifi27l2a)* (*Ncf1^-/-^* 6.46-fold increase), IFN-γ response gene *Oas2* (*Ncf1^-/-^* 3.19-fold increase), IL-6-Jak-Stat3 signaling downstream gene *Cd14* (*Ncf1^-/-^* 2.12-fold increase)and inflammatory gene *Nos2* (*Ncf1^-/-^* 8.26-fold increase) expression with quantitative PCR (qPCR). Consistently, these genes were significantly upregulated in activated *Ncf1^-/-^* neutrophils from joints (**Figure 2C**). These data indicates that activated *Ncf1^-/-^* neutrophils from joints were the source of pro-inflammatory characteristics in serum-induced arthritis. In addition, gene set enrichment analysis (GSEA) and network analysis showed interacting enriched gene sets in phenotype *Ncf1^-/-^* are associated with inflammation (**Figure 3A**). Genes associated with those pro-inflammatory pathways, including type I (**Figure 3B**) and II IFN responses (**Figure 3C**), IL-6-Jak-Stat3 signaling (**Figure 3D**) and TNF-α signaling *via* NF-κB (**Figure 3E**) were up-regulated in *Ncf1^-/-^* neutrophils. The enrichment plots of those results are shown in **Supplementary Figure 2**. Taken together, our data point out that these immune-complex activated Nox2-deficient neutrophils had distinct transcriptional profiles with enhanced pro-inflammatory characteristics.

### NOX2-Deficient Neutrophils From Inflammatory Joints Were Less Suppressive for T Cells Proliferation *In Vitro*

**Figure 4:**
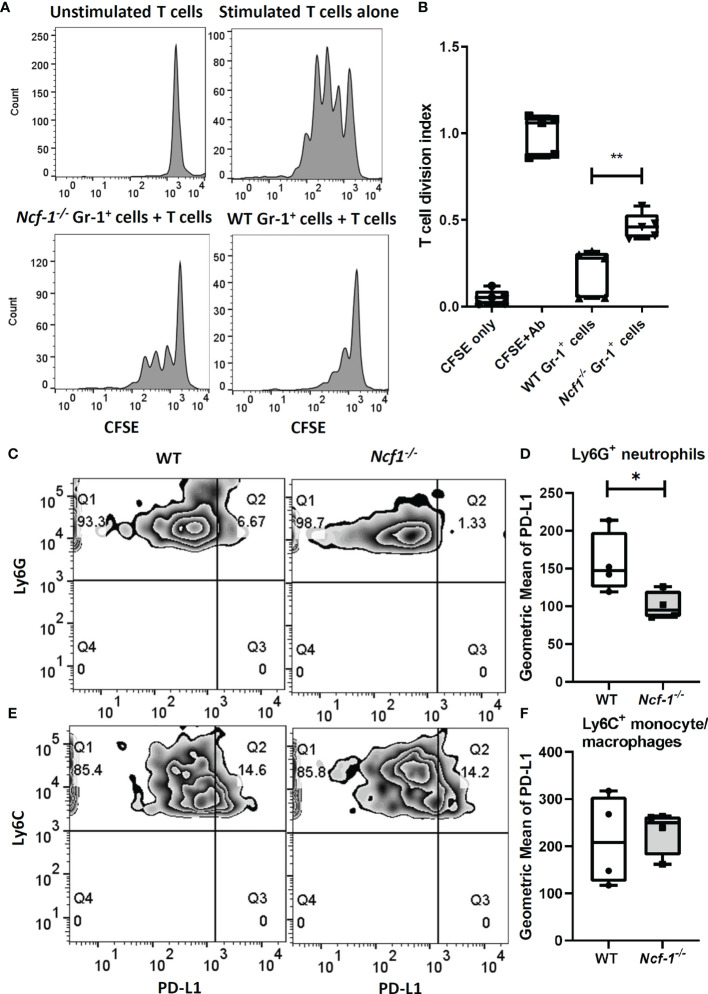
NOX2-deficient neutrophils have less suppressive activity and lower PD-L1 expression. CFSE-labelled total WT T cells stimulated with anti-CD3, anti-CD28 Abs were cultured alone or co-cultured with WT or NOX2-deficient Gr-1^+^ cells isolated from arthritic joints for 3 days. (WT, n = 5; *Ncf1^-/-^*, n = 5). **(A)** Representative gating. **(B)** The division index were calculated by using proliferation modelling in FlowJo V10 software. Analysis of PD-L1 expression level on Ly6G^+^ neutrophils **(C)** and Ly6C^+^ monocytes/macrophages **(E)** from arthritic joints of WT and *Ncf1 ^-/-^* mice were determined by flow cytometry. Quantitative geometric MFI are shown in **(D, F)**. All measurements were plotted by using Prism. The statistically significant differences between groups are indicated with *, **, (*p < 0.05, **p < 0.01). The experiment was repeated twice with similar results.

To investigate whether those accumulated WT and NOX2-deficient neutrophils differ in their immunosuppressive function (8) in addition to different pro-inflammatory gene expression, we established an *in vitro* suppressive assay to test the activity of neutrophils in suppressing T cell proliferation. Gr-1^+^ cells from inflamed joints of both WT and *Ncf1^-/-^* mice on day 7 were collected, and co-cultured with WT splenic T cells stimulated with anti-CD3, anti-CD28 antibodies. The result of flow cytometry showed that the proliferation of WT splenic T cells was suppressed to about 20% of the control level when they were co-cultured with WT arthritic neutrophils. The neutrophil-mediated suppression was weaker when NOX2-deficient arthritic neutrophils were used in the co-culture experiments. We found that the proliferation of WT splenic T cells were suppressed to about 45% the control level when they were co-cultured with NOX2-deficient arthritic neutrophils (**Figures 4A, B**). These data suggest that the immunosuppressive function of NOX2-deficient neutrophils is impaired.

### Reduced PD-L1 Expression on NOX2-Deficinet Ly6G^+^ Neutrophils May Contribute to More Severe Inflammatory Arthritis

Based on the previous reports that neutrophils can modulate T cells response through PD-L1 (28) and PD-L1 expression could be regulated by induced ROS (29), we measured the level of PD-L1 on myeloid cells including Ly6G^+^ neutrophils and Ly6C^+^ monocytes/macrophages from arthritic tissues of WT and *Ncf1^-/-^* mice with flow cytometry. Interestingly, the expression of PD-L1 on Ly6G^+^ neutrophils from *Ncf1^-/-^* inflamed joints was decreased when compared with that on neutrophils from WT controls. (Average MFI of *Ncf1^-/-^* and WT were 100 and 157 respectively) (**Figures 4C, D**). In contrast, level of PD-L1 on Ly6C^+^ monocytes/macrophages from inflamed joints of *Ncf1^-/-^* and WT mice had no significant difference (**Figures 4E, F**). These data hence suggest that lower level of PD-L1 on the dominant Ly6G^+^ neutrophils may contribute to the severity of arthritis in NOX2-deficient mice.

### Recombinant PD-L1-Fc Treatment Lowered Severity of Arthritis and Cytokine Expressions in *Ncf1^-/-^* Mice

**Figure 5:**
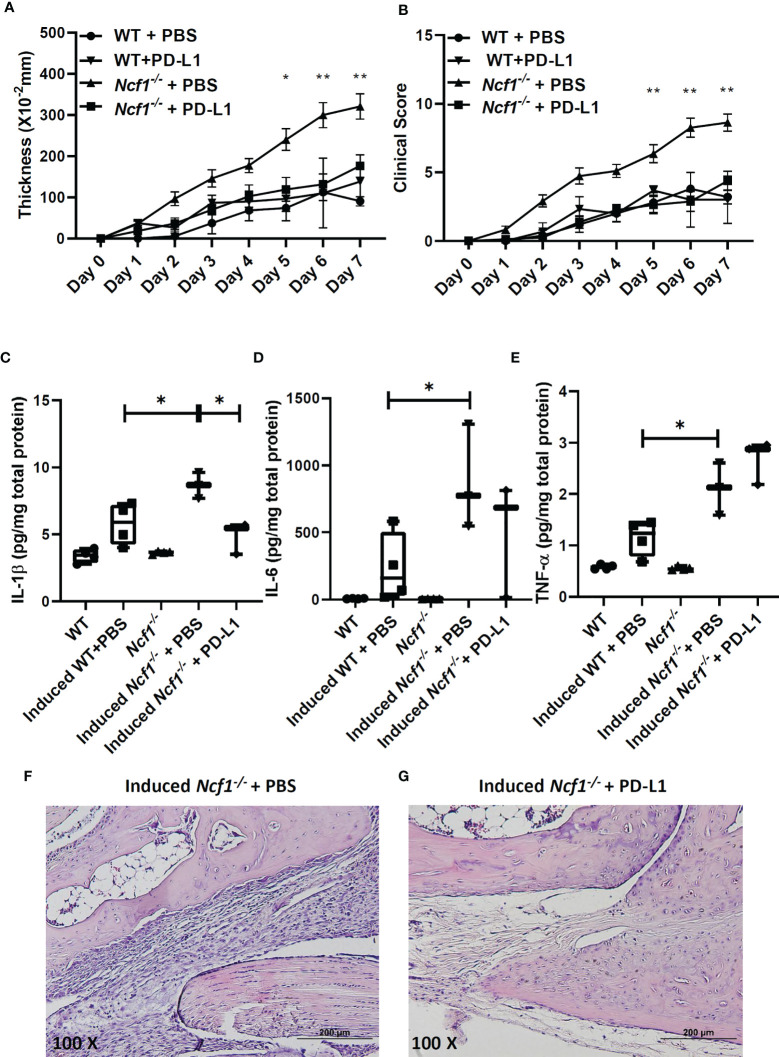
Recombinant PD-L1-Fc treatment reduced severity and pro-inflammatory cytokine expression in *Ncf-1^-/-^* arthritic joints. The effects of recombinant PD-L1-Fc on the thickening **(A)** and clinical scores **(B)** of arthritis in WT and *Ncf1* ^−^ *^/^* ^−^ mice are shown (mean ± SEM). Induced WT + PBS, n = 5; Induced WT + PD-L1-Fc, n = 3; Induced *Ncf1 ^-/-^* + PBS, n = 11; Induced *Ncf1 ^-/-^* + PD-L1-Fc, n = 8. Wrist homogenates from non-induced WT (N=4), *Ncf1^-/-^* (N=4), induced WT(N=4), *Ncf1^-/-^* treated with PBS (N=3) and induced *Ncf1^-/-^* mice treated with recombinant PD-L1-Fc (N=3) were quantified for **(C)** IL-1β, **(D)** IL-6, **(E)** TNF-α with Luminex assay. The typical histopathological images of H&E-stained joints from induced *Ncf1^-/-^* mice treated with PBS **(F)** and induced *Ncf1*−*/*− mice treated with PD-L1-Fc **(G)** are shown. Micrographs were representative of 3-4 mice in each group. Bar = 200 µm (original magnification 100X). The statistically significant differences between groups are indicated with *, **, (*p < 0.05, **p < 0.01). The experiment was repeated twice with similar results.

We went on to try boosting PD-L1-PD-1 pathway to ameliorate the serum-induced arthritis by treating *Ncf1^-/-^* arthritic mice with recombinant PD-L1-Fc. The recombinant PD-L1-Fc was injected into WT and *Ncf1^-/-^* arthritic mice on day 0, 2, 4, 6 and the swelling and clinical score were measure for 7 days. As shown in **Figures 5A, B**, recombinant PD-L1-Fc treatment reduced joints swelling and clinical scores of *Ncf1^-/-^* arthritic mice when compared with PBS treated controls. Moreover, there was no significant difference of severity in induced WT mice after PD-L1-Fc treatment. (**Figures 5A, B**). Next, we analyzed the cytokine profiles in joints of non-induced WT, *Ncf1^-/-^*, induced WT, *Ncf1^-/-^*mice and induced *Ncf1^-/-^*mice after treatment with recombinant PD-L1-Fc. Consistent with our previous report, expressions of IL-1β (**Figure 5C**), IL-6 (**Figure 5D**) and TNF-α (**Figure 5E**) were significantly higher in induced *Ncf-1^-/-^* joints than those in induced WT joints. After PD-L1-Fc treatment, IL-1β was lowered by about 56% (P value= 0.0134) in induced *Ncf-1^-/-^* joints, and a declined trend was shown in IL-6 expression while TNF-α expression was not significantly affected. Additionally, we found that immune cell infiltration is markedly decreased in PD-L1 Fc treated joints when compared to PBS control (**Figures 5F, G**). These data indicate that boosting PD-L1 axis may ameliorate inflammatory arthritis in NOX2-deficient condition.

In conclusion, NOX2-deficient neutrophils with higher pro-inflammatory characteristics and lower anti-inflammatory function contribute to the enhanced joint inflammation in K/BxN serum-transfer arthritis in NOX2-deficient mice.

## Discussion

A growing body of evidence supports important regulatory roles of leukocytes-derived ROS in inflammatory arthritis (21, 22, 30). In our previous finding, NOX2-deficient mice developed enhanced inflammatory arthritis. Moreover, neutrophils were abundantly accumulated in NOX2-deficient arthritic joints (21). In this study, we showed that gene expression profile of NOX2-deficient arthritic neutrophils pinpointed type I and II IFN responses and TNF-α, IL-6 signaling pathways as the upregulated pro-inflammatory mechanisms. Moreover, the lowered PD-L1 expression on NOX2-deficient neutrophils may contribute to the defective immune checkpoint regulatory function of these cells. The hitherto unclear pathogenic role of NOX-2 deficient neutrophils in tissue inflammation was clarified in this study. In summary, we found that health and functions of neutrophils are impaired due to NOX2 deficiency, which eventually lead to enhanced joint inflammation in K/BxN arthritis in NOX2-deficient mice.

NOX2 complex is composed of gp91phox, p47 phox, p22 phox, p67 phox and p40 phox. The phosphorylation of p47 phox, encoded by Ncf1 gene, recruits other subunits from the cytosol to bind and form NOX2 complex. NOX2 complex now is capable of transferring an electron from cytosolic NADPH to extracellular oxygen for producing ROS. When *Ncf1* gene or genes of other significant components of NOX2 complex are disrupted, NOX2 complex losses the ability to produce ROS (11). The local inflammatory responses of NOX2 vary in different organs and different stimulations (31, 32). In this study, we focused on NOX2 regulation of joint inflammation (a local inflammation). However, NOX2 deficiency can also regulate systemic inflammation. It has been shown that NOX2 plays a protective role in SLE, a systemic autoimmune disease. NOX2-deficient lupus-prone mice had significantly aggravated lupus (33). Moreover, NOX2-deficient mice exhibit severe thymic atrophy, lymphopenia and increased neutrophilic inflammation in a zymosan-induced systemic inflammation (34). Thus, NOX2 deficiency may lead to systemic inflammation and some local inflammation including arthritis. In addition, it has been reported that depletion of p22phox with siRNA led to a significant decrease in mitochondrial ROS generation (35). The indirect interaction between NOX2 activity and mitochondrial ROS production may further lower the cellular ROS levels. Deficiency in local and systemic ROS production caused by defective NOX2 hence may underlie our observation that more severe arthritis was induced in *Ncf1^-/-^* mice.

Neutrophils have been recognized as the main players in host defense against microorganisms and an essential role in the innate immunity. Many studies addressed the damaging role of neutrophils by releasing tissue-damaging molecules including proteases or molecules that can promote inflammation including chemoattractants or cytokines (36). These microbiocidal and tissue-damaging mechanisms, however, cannot account for the more severe immune-mediated tissue inflammation in NOX2-deficient animals (15, 16, 21, 22, 37) and in patients with CGD (38–40). In this study, we detected *IL1b*, *Cxcl2, Cxcl3*, *Cxcl10* and *Mmp3* up-regulation in NOX2-deficient neutrophils from arthritic joints. Neutrophils require Cxcl2 for more neutrophils recruitment (41). Inhibition of Cxcl3 in a mouse model of RV-induced exacerbation of asthma decreased the accumulation of Cxcr2^+^ neutrophils (42). Cxcl10-Cxcr3 acts in an autocrine way on the oxidative burst and chemotaxis in the inflamed neutrophils (43). Mmp3 is actively involved in joint destruction in RA patients, and it degrades collagen types II, III, IV, IX and X, proteoglycans, fibronectin, laminin, and elastin (44). It is possible that NOX2-derived ROS modify the signaling molecules in the pathways that induce pro-inflammatory mediators (45). In addition, ROS may drive epigenetic changes including modification of DNA bases and histones (46, 47). Moreover, immune cells trafficking can be affected by ROS as well (48, 49).

Research of neutrophil is not only to investigate the pro-inflammatory roles, but also the regulatory functions (50). Its role in modulation of adaptive immune responses, for example suppressing T cell-mediated immune responses, was originally identified in a murine model of cancer that Gr-1^+^ cells can inhibit T cell activation through CD3/CD28 co-stimulation (51, 52). Our finding suggest that those accumulated NOX2-deficient neutrophils have impaired suppressive function (anti-inflammation). Without suppressive function, in addition to the pro-inflammatory role, NOX2-deficient neutrophils dominantly contribute to development of more severe serum-induced arthritis.

We aimed to study both pro-inflammatory and anti-inflammatory roles of neutrophils. Since our RNA-seq data revealed pro-inflammatory TNF-α and IL-6 -JAK-STAT3 pathways, which are routinely treatable with biologics and small-molecule drugs in clinical settings, we went on to analyze the anti-inflammatory immune checkpoint molecules of PD-L1.Targeting PD-1 or PD-L1 to block immune checkpoints have been shown to be beneficial for treatment of several human cancers (53). However, PD-1-PD-L1 blocking has been reported to cause various autoimmune or inflammatory diseases including inflammatory arthritis (54). In this study, we found that expressions of PD-L1 was lower on NOX2-deficient Ly6G^+^ neutrophils, while that expression on Ly6C^+^ monocytes/macrophages had no significant difference (**Figure 4**). Although we did not detect significant differential gene expressions of PD-L1 in our RNA-seq data, it has been known that the regulation of PD-L1 expression is complex. Also, it has been noted that the relationship between transcriptional levels and protein expressions is not consistent in neutrophils (55). Post-translational regulation has been reported to be important for the cellular expression and function of PD-L1 (56, 57). Furthermore, since neutrophils are the dominant immune cell population that infiltrated into joints at initial stage of arthritis, we propose that the lower PD-L1 expressions on NOX2-deficient neutrophils may contribute to the development of more severe serum-induced arthritis. Similar to previous reports showing that administration of PD-L1-Fc can inhibit the development of collagen-induced arthritis (CIA) (54, 58), our results showing the effectiveness in PD-L1-Fc in ameliorating serum-induced arthritis in NOX2-deficient mice further support the critical role of PD-1/PD-L1 immune regulation in joint inflammation.

Redox regulation has been proposed to be important in immune regulation (59, 60). Several studies have indicated that lack of phagocytes-produced ROS enhances arthritis susceptibility and severity (15, 21, 22, 61). We previously reported that treating NOX2-deficient arthritic mice with Anakinra, a recombinant human interleukin-1 receptor antagonist, reduced severity of arthritis at certain level (21). In this study, our findings suggest that PD-L1 treatment significantly decrease IL-1β level in arthritic joints (**Figure 5**) and control inflammation, therefore PD-L1 treatment may be the potential treatment for arthritic patients. Our results from the PD-L1 experiments not only support the role of weakened immune checkpoint regulation in the more severe arthritis in NOX2-deficient conditions, but also showed that replenishment of PD-L1 may be a potential therapeutic approach in some patients with immune-mediated arthritis.

This study for the first time revealed the arthritogenic role of NOX2-deficient neutrophils on the initiation and progression of immune-mediated arthritis. The understanding of redox-sensitive mechanisms underlying the increased pro-inflammatory and compromised immune-regulatory cellular activities of neutrophils promises to improve the treatment for patients with neutrophil-dominant inflammatory arthritis.

## Data Availability Statement

The original contributions presented in the study are publicly available. This data can be found here: https://www.ncbi.nlm.nih.gov/bioproject/PRJNA753258.

## Ethics Statement

The animal study was reviewed and approved by Laboratory Animal Center, College of Medicine, National Cheng Kung University.

## Author Contributions

C-CS initiated the idea. Y-CL, P-CC, and C-CS designed the experiments. Y-CL, S-YW, P-CL and T-YC performed the experiments. Y-CL and P-CC analyzed the data. Y-FH, C-AC, C-HW, C-CH and C-LY provided critical materials and reagents for the study. Y-CL, P-CC, and C-CS wrote and edited the manuscript. All authors contributed to the article and approved the submitted version.

## Funding

This work was supported by Ministry of Science and Technology, Taiwan (MOST109-2314-B-006-049).

## Conflict of Interest

The authors declare that the research was conducted in the absence of any commercial or financial relationships that could be construed as a potential conflict of interest.

## Publisher’s Note

All claims expressed in this article are solely those of the authors and do not necessarily represent those of their affiliated organizations, or those of the publisher, the editors and the reviewers. Any product that may be evaluated in this article, or claim that may be made by its manufacturer, is not guaranteed or endorsed by the publisher.

## Abbreviations

CGD, chronic granulomatous disease; IL, interleukin; MPO, myeloperoxidase; NOX2, NADPH oxidase 2; RA, rheumatoid arthritis; ROS, reactive oxygen species; TNF-α, tumor necrosis factor-α; IFN, interferon; NF-κB, Nuclear Factor Kappa B; Ncf1, Neutrophil Cytosolic Factor 1; PD-L1, Programmed death-ligand 1; JAK, Janus kinase; STAT3, signal transducer and activator of transcription 3; MFI, mean fluorescence intensity; FDR, false discovery rate; H&E, hematoxylin and eosin; WT, wild type; GSEA, gene set enrichment analysis; PMA, Phorbol 12-myristate 13-acetate.
