# Supplementary material for: NOX2-Deficient Neutrophils Facilitate Joint Inflammation Through Higher Pro-Inflammatory and Weakened Immune Checkpoint Activities
Source: Front Immunol. 2021 Sep 7;12:743030. doi: 10.3389/fimmu.2021.743030 (PMC8452958; doi:10.3389/fimmu.2021.743030)
Supplement: Supplementary file 1 [file DataSheet_1.docx]

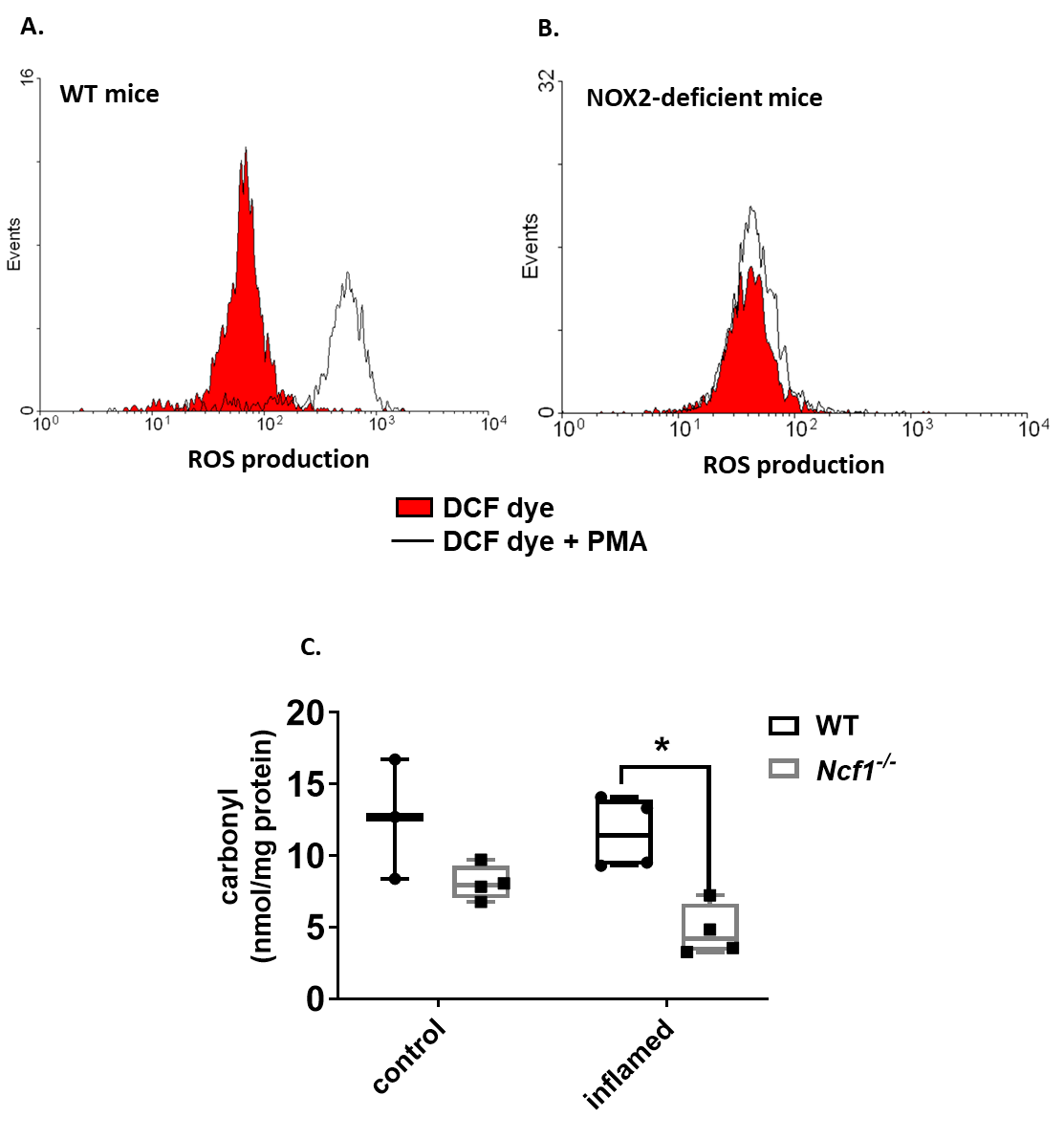


**Supplementary Figure 1 Decreased ROS level were detected in NOX2-deficient peripheral leukocytes and joint tissues.**

ROS production was determined in (**A**) WT and (**B**) NOX2-deficient peripheral blood leukocytes by flow cytometry. Quantitative data of protein carbonylation in control and inflamed joint tissues was shown in (**C**). All measurements were plotted by using Prism. The statistically significant differences between groups are indicated with *, **, and *** (*p < 0.05, **p < 0.01, ***p < 0.001). The experiment was repeated twice with similar results.
