# Supplementary material for: NOX2-Deficient Neutrophils Facilitate Joint Inflammation Through Higher Pro-Inflammatory and Weakened Immune Checkpoint Activities
Source: Front Immunol. 2021 Sep 7;12:743030. doi: 10.3389/fimmu.2021.743030 (PMC8452958; doi:10.3389/fimmu.2021.743030)
Supplement: Supplementary file 2 [file DataSheet_2.docx]

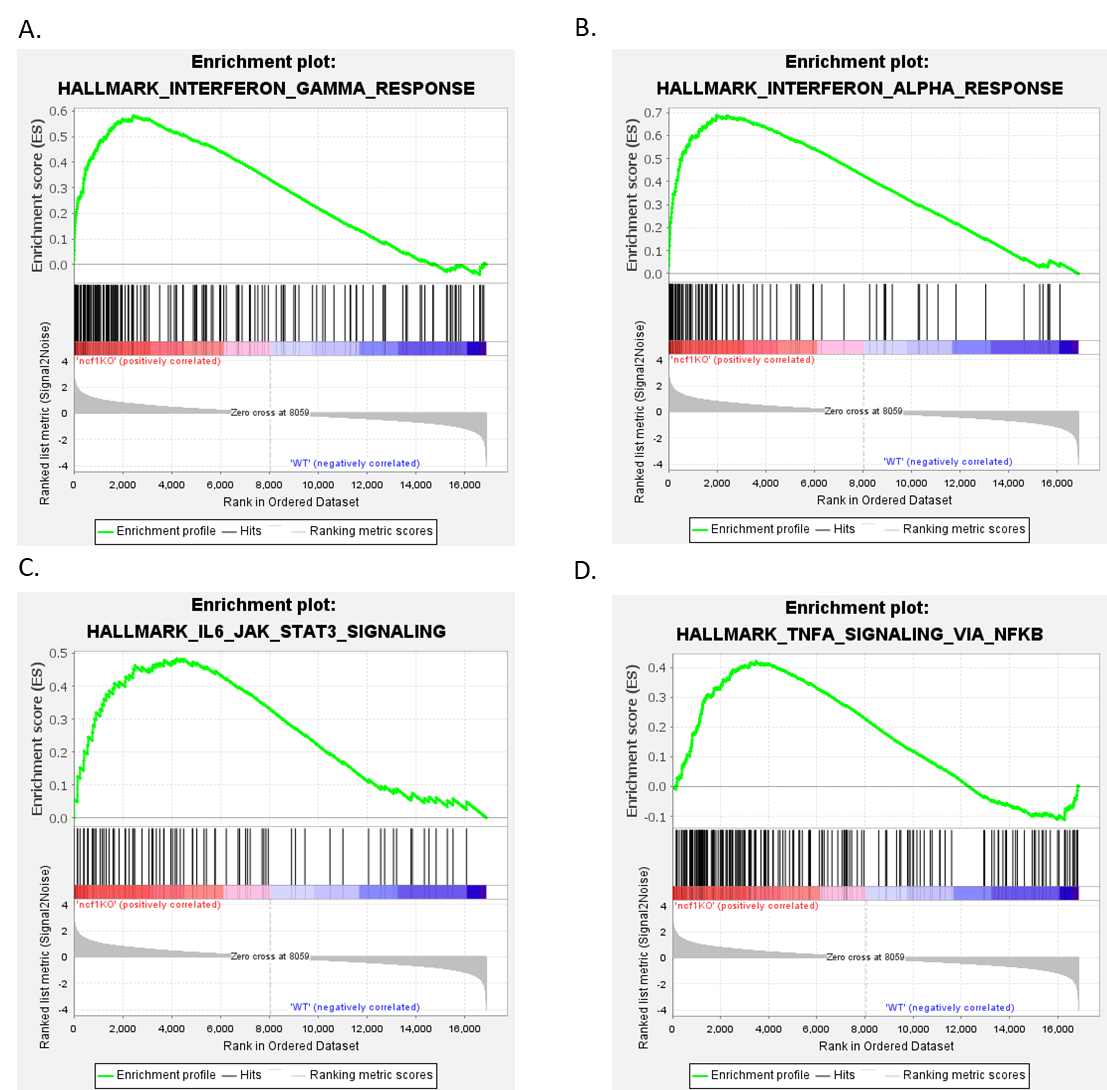


**Supplementary Figure 2 Enrichment plots from GSEA**

Enrichment plots from GSEA show that genes enriched in *Ncf1^-/-^* neutrophils correlates with (**A**) IFNγ response, (**B**) IFNα response, (**C**) IL-6-JAK-STAT3 signalling and (**D**) TNF-α signalling via NF-κB pathways.
